# Data on the effect of a muscimol treatment in caspase activation in descending neurons of lampreys after a complete spinal cord injury

**DOI:** 10.1016/j.dib.2018.11.003

**Published:** 2018-11-06

**Authors:** Daniel Sobrido-Cameán, María Celina Rodicio, Antón Barreiro-Iglesias

**Affiliations:** Department of Functional Biology, CIBUS, Faculty of Biology, Universidade de Santiago de Compostela, 15782 Santiago de Compostela, Spain

## Abstract

In this article, caspase activation in identifiable reticulospinal neurons of lampreys was inhibited after a complete spinal cord injury using a specific agonist of the GABAA receptor (muscimol). The data presented in this article are quantifications of fluorescent labelling of identifiable descending neurons of larval lampreys after a complete spinal cord injury using fluorochrome-labelled inhibitors of caspases (FLICA) and the corresponding statistical analysis. A single dose of muscimol decreased the intensity of FLICA labelling in giant identifiable reticulospinal neurons following spinal cord injury in lampreys.

**Specifications table**

**Table:** 

Subject area	Neuroscience
More specific subject area	Regenerative biology
Type of data	Graph, Figure, Table
How data was acquired	Confocal microscope (TCS-SP2; Leica, Wetzlar, Germany)
Data format	Analysed data, processed data.
Experimental factors	Larval sea lampreys were treated with muscimol after a complete spinal cord injury. Caspase activation was analysed in identifiable descending neurons using fluorochrome-labelled inhibitors of caspases (FLICA).
Experimental features	The effect of muscimol on caspase activation after a complete spinal cord injury was analysed using fluorescence microscopy.
Data source location	Department of Functional Biology, Faculty of Biology, CIBUS, Universidade de Santiago de Compostela
Data accessibility	The data are available within this article.
Related research article	Romaus-Sanjurjo et al. [1]

**Value of the data**

• Fluorochrome-labelled inhibitors of caspases allow the detection of changes in caspase activation.
• This dataset is of interest for those studying signalling pathways modulating neuronal survival after spinal cord injury in fishes.
• This dataset is of interest for the development of neuroprotectants as a therapy for spinal cord injury.

## 1 Data

**Fig. 1:**
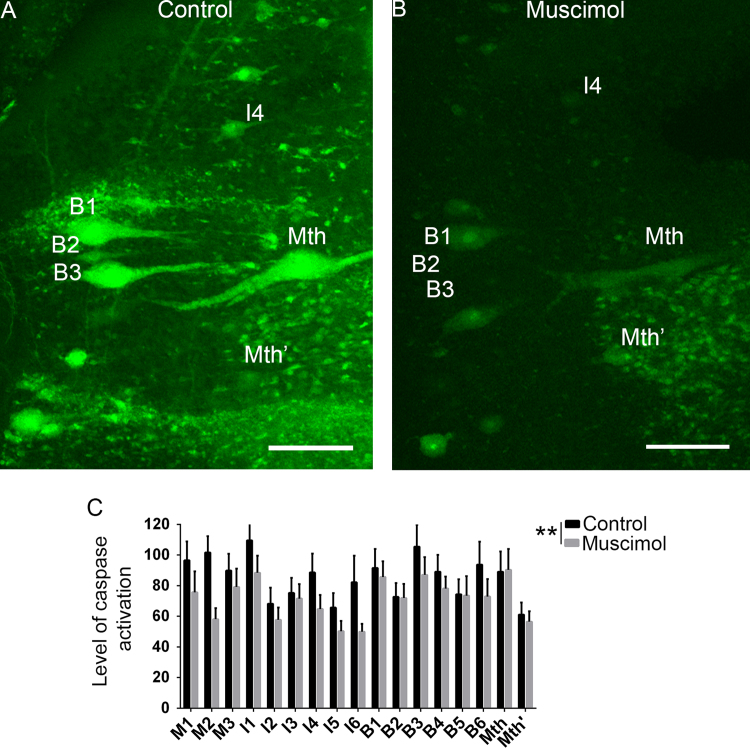
Muscimol treatment inhibits caspase activation in identifiable descending neurons. A: Photomicrograph of a whole-mounted brain showing identifiable descending neurons with intense FLICA labelling in control animals. B: Photomicrograph of a whole-mounted brain showing identifiable descending neurons with a reduction in the intensity of FLICA labelling in muscimol treated animals. C: Graph showing a significant change (Mann Whitney *U*-test, *p* = 0.0054; asterisks) in the level of caspase activation (intensity of fluorescent FLICA labelling; *Y* axis) after the muscimol treatment in identifiable descending neurons (*X* axis). Rostral is up and the ventricle to the left in all photomicrographs. Scale bars: 150 µm.

Recently, our group reported that endogenous GABA promotes axonal regeneration of identifiable reticulospinal neurons after a complete spinal cord transection in lampreys [1]. The beneficial effect of GABA appears to be caused by a reduction in the activation of caspases through the activation of GABAB receptors [1]. Other authors also reported that increased GABAergic inhibition trough GABAA receptors is related to a better recovery of function following spinal cord injury in lampreys [2]. Here, we show that the activation of GABAA receptors after a complete spinal cord transection also inhibits caspase activation in identifiable reticulospinal neurons of lampreys. A treatment with a single dose of muscimol, which is a specific agonist of the GABAA receptor, following a complete spinal cord transection reduced the activation of caspases in giant reticulospinal neurons of lampreys as revealed by a decreased fluorescent intensity of FLICA labelling (Fig. 1). Table 1 shows the mean ± S.E.M. of fluorescence intensity of each neuronal type and the number of identifiable neurons used for the statistical analysis. The data from each individual neuron is given in Table S1.

**Table 1:** Table showing the total number of identifiable reticulospinal neurons that were included in the analyses and the mean ± S.E.M. of fluorescence intensity of FLICA labelling of each identifiable neuron. The data from each individual neuron is given in Table S1.

	**Control**			**Muscimol**		
	**Mean**	**S.E.M.**	***N***	**Mean**	**S.E.M.**	***N***
**M1**	96.53958	± 12.3778	12	75.67378	± 13.63257	14
**M2**	101.704	± 10.53632	12	58.14621	± 7.223279	14
**M3**	89.88461	± 10.92918	13	79.14021	± 12.03266	14
**I1**	109.5599	± 15.80085	12	88.42269	± 11.18598	16
**I2**	68.21775	± 10.45724	12	57.74719	± 7.989043	16
**I3**	75.28416	± 9.889297	13	71.58913	± 9.475986	16
**I4**	88.61723	± 12.39366	13	64.75288	± 9.115862	16
**I5**	65.69509	± 9.503008	11	50.27394	± 6.789322	16
**I6**	82.24782	± 17.39615	11	49.91087	± 5.125011	16
**B1**	91.59154	± 12.3856	15	85.62478	± 10.22685	18
**B2**	72.6452	± 9.034819	15	71.90128	± 9.277125	18
**B3**	105.5087	± 14.23046	15	87.06628	± 11.55049	18
**B4**	89.14333	± 10.9955	15	78.09394	± 7.780739	18
**B5**	74.33907	± 9.773049	15	73.49995	± 12.7075	18
**B6**	93.76653	± 14.91869	15	72.91844	± 11.33416	18
**Mth**	89.1006	± 13.2872	15	90.32294	± 13.61253	18
**Mth׳**	61.15147	± 7.887609	15	56.45844	± 6.828169	18

## 2 Experimental design, materials, and methods

### 2.1 Animals

All experiments involving animals were approved by the Bioethics Committee at the University of Santiago de Compostela and the *Consellería do Medio Rural e do Mar* of the *Xunta de Galicia* (License reference JLPV/IId; Galicia, Spain) and were performed in accordance to European Union and Spanish guidelines on animal care and experimentation. Animals were deeply anaesthetized with 0.1% MS-222 (Sigma, St. Louis, MO) in lamprey Ringer solution (137 mM NaCl, 2.9 mM KCl, 2.1 mM CaCl^2^, 2 mM HEPES; pH 7.4) before all experimental procedures and euthanized by decapitation at the end of the experiments.

Mature and developmentally stable larval sea lampreys, *Petromyzon marinus* L. (*n* = 17; between 95 and 120 mm in body length, 5–7 years of age), were used in the study. Larval lampreys were collected from the river Ulla (Galicia, Spain), with permission from the *Xunta de Galicia)* and maintained in aerated fresh water aquaria at 14–20 °C with a bed of river sediment until their use in experimental procedures. Lampreys were randomly distributed between the different experimental groups.

### 2.2 Spinal cord injury surgical procedures

Complete spinal cord transections were performed as previously described [3]. The rostral spinal cord was exposed from the dorsal midline at the level of the 5th gill by making a longitudinal incision with a scalpel (#11). A complete spinal cord transection was performed with Castroviejo scissors and the spinal cord cut ends were visualized under the stereomicroscope. Animals with a complete spinal cord transection were assigned to either a vehicle treated control group (*n* = 8) or to muscimol treated group (*n* = 9). After spinal transections, the animals were returned to fresh water tanks. The animals were allowed to recover in individual fresh water tanks at 19.5 °C. Animals were analysed 2-weeks post-lesion (wpl). The experiment was carried out in 3 different batches of animals.

### 2.3 Drug treatments

Muscimol was dissolved in distilled water at a concentration of 25 µM, soaked in a small piece of Gelfoam (Pfizer; New York, NY) and placed on top of the site of injury at the time of transection as previously described [4]. Gelfoam soaked in distilled water served as a control.

### 2.4 Detection of activated caspases in whole-mounted brain preparations

The Image-iT LIVE Green Poly Caspases Detection Kit (Cat. No. I35104, Invitrogen, USA) was used to detect activated caspases in identifiable descending neurons (the M1, M2, M3, I1, I2, I3, I4, I5, I6, B1, B2, B3, B4, B5, B6, Mth and Mth’ neurons) of larval sea lampreys 2 weeks after the complete spinal cord transection. This kit contains 1 vial (component A of the kit) of the lyophilized FLICA reagent (FAM-VAD-FMK). The reagent associates a fluoromethyl ketone (FMK) moiety, which can react covalently with a cysteine, with a caspase-specific aminoacid sequence [valine-alanine-aspartic acid (VAD)]. A carboxyfluorescein group (FAM) is attached as a fluorescent reporter. The FLICA reagent interacts with the enzyme active centre of an activated caspase via the recognition sequence, and then attaches covalently through the FMK moiety. Experiments for the detection of activated caspases in whole-mounted brain preparations using FLICA labelling were done as previously described [5].

### 2.5 Imaging and quantifications

The quantification of the intensity of FLICA labelling was done as previously described [6]. Briefly, photomicrographs were acquired with a spectral confocal microscope (model TCS-SP2; Leica, Wetzlar, Germany). Images were always acquired under the same microscope conditions for control and treated animals. Quantification of mean fluorescent intensity (mean grey value) of each identifiable neuron was done using the Fiji software [7]. The mean of fluorescence intensity of each type of identifiable descending neuron was used for statistical analyses. Figure plates were generated using Adobe Photoshop CS6 (Adobe Systems).

### 2.6 Statistical analyses

Statistical analysis was carried out using Prism 6 (GraphPad software, La Jolla, CA). Data are presented as mean ± S.E.M. Normality of the data was determined by the Shapiro-Wilk, D׳Agostino and Pearson omnibus and Kolmogorov–Smirnov normality tests. All data passed all the normality tests. The results of control versus treatment groups were analysed by Mann-Whitney *U*-test.
